# Observation of upstream particle movement without the involvement of the marangoni effect

**DOI:** 10.1371/journal.pone.0317312

**Published:** 2025-03-28

**Authors:** Aryan Hussain Sahir, Saiham Saif Emon

**Affiliations:** Jhenaidah Cadet College, Jhenaidah, Bangladesh; Manipal Academy of Higher Education, INDIA

## Abstract

Upstream Contamination is a counter-intuitive phenomena of fluid dynamics, where particles can go against the liquid stream and climb, higher containers. Previous studies have attributed the movement of particles against the flow of water to the Marangoni effect, where surface tension gradients drive fluid movement. However, the effect was not enough to account for the motion of particles. Meanwhile, this study challenges that explanation by documenting the ascent of fine particles of Iron Fillings, through a water jet where the lower container exhibited higher surface tension due to an aqueous calcium chloride (CaCl_2_) solution. Contrary to the Marangoni effect, which predicts that particles should move from areas of lower to higher surface tension, our observations showed that the particles can move upwards even from a higher surface tension to a lower surface tension region. Afterward, other factors influencing this phenomenon were studied, including the height of the upper container from the lower one, the angle between the channel and the horizontal axis, temperature, and surface tension gradient. Each of them, suggests that factors other than surface tension gradients, such as fluid dynamics and turbulence, play a significant role in particle behavior in these conditions. This study can help us understand how some *‘safe-ecosystems’, cell mechanisms and medicine production* can be contaminated in an unthinkable way so that these contaminators can be prevented and a safer ecosystem and medicare development can be ensured and steps to safeguard protoplasm from harmful contaminants can be taken.

## Introduction

The Marangoni effect, discovered in the 19th century, describes fluid flow resulting from surface tension gradients. Observations of particles moving against the expected flow of water have often been attributed to this effect [1,2]. For instance, Sebastian Bianchini of the University of Havana observed tea leaves moving against the flow when hot water was poured into a cup, and similar observations have been reported with chalk-contaminated water. These incidents were interpreted as being driven by the Marangoni effect, which drives particles from regions of lower to higher surface tension. Although, it was observed that the motion of the particles couldn’t alone be explained by the described effect [1].

Meanwhile, this experiment was primarily conducted to judge the contribution of the effect in the motion of the particles against the stream, to see whether the particles could climb up the jet even without the involvement of the effect. To do so, the lower container was filled with a higher surface tension solution than that of the upper one, and then the experiments were conducted to see whether the upstream contamination phenomenon could occur in such a scenario.

If detected, then the observations that are made in this paper are going to establish the effect only as a *“supporting factor”* and *not the main driving cause.* This will help the scientific community to identify what are the main driving force behind the particles’ struggle against the water downstream water jet.

Again, through several continuous experiments, a hypothesis is that the particles can climb up through the jet when the no-slip boundary layer condition is such that, the particles get enough drag and buoyancy force to penetrate through the downstream water while defying the force of gravity. Even though this ***shall not be the primary focus*** of the investigation, the primary focus will be to learn whether the particles can go upstream without the support of the Marangoni Effect [3].

Understanding the real causes behind this phenomenon *might* provide us with support even up to the microscopic levels. Protoplasm, the living portion of the cell, takes part in different biological mechanisms, like nutrient transport, waste removal, etc. Among these, all require the support of fluid dynamics, which poses the threat that during one of the mechanisms, harmful particles may infiltrate into the protoplasm, through the phenomenon of upstream contamination, brownian motion and others. Thus, it’s highly important to understand this phenomenon to learn more about the possible risks that our living quanta face.

## Literature review

Since the discovery of the phenomenon of upstream Contamination in 2008, the researcher *Bianchini et al. (2013)*, in their research paper have largely attributed the movement of particles to the Marangoni Effect. But in their paper, they also admitted that the Marangoni Effect doesn’t explain the particle’s movement alone. Which primarily led us to investigate whether the effect is the main cause behind the phenomenon.

The property of the Marangoni Effect to transfer mass along the surface tension gradient, to a higher surface tension has been known since the work of *Carlo Marangoni (1865)*, which was later proved in a more sophisticated way by Troian et al. (1990), which studied Marangoni Effect in a more experimented way. These experiments have established the fact that the Marangoni Effect can only transfer mass from lower to higher surface tension liquid, to a great extent. Thus, if some phenomenon is observed otherwise, then it can not be attributed to the effect, which will be the prime focus of this investigation.

The hypothesis regarding the Boundary Layer Effect is also somewhat supported by the works of Schlichting and Gersten (2016), which describe the different behaviors of the *Boundary Layer Effect, Laws of Friction, Shear Force, Effect of Angle on Friction*, etc. This work is going to support this experiment to a great extent because the hypothesis shall be checked with investigations into many a factor, which is described in the work of Schlichting and Gersten (2016). Furthermore, the works of Batchelor (1967), also discuss the hypothesis regarding the effect of Buoyancy and drag force upon particles, which has been thoroughly investigated in the paper.

Again, the work of Williamson (1996), has been thoroughly studied since it describes how velocity gradient and turbulent flow effects the formation of vortexes. Vortexes have been also studied with suspicion by the initial researchers of Upstream Contamination, Bianchini et al. (2013).

The work of Bormashenko (2015) and many others have studied the property of denser particles to float above liquid due to the surface tension of it, which inspired the investigation to use iron powder, since it was able to float on water without changing the surface tension when added.

This body of evidence with correspondence with the conducted investigations, collectively points to a multi-faceted explanation for upstream contamination, where the Marangoni effect acts as a supporting rather than a primary factor. Instead, the key mechanisms involve the boundary layer effect, drag force, and buoyancy, along with the physical properties of the contaminant particles.

## Materials

The materials that were used in the experiment were chosen due to some specific reasons and after vigorous testing, the prime ones are-

1. **Iron powder:** The experiment utilized finely ground pure iron fillings from the American Heritage Company. This fine powder allowed the surface tension of the CaCl₂ solution to support the iron particles, enabling them to float on the water despite being denser [4]. Additionally, iron powder was selected because it did not affect the surface tension of the CaCl₂ solution during the short duration of the experiment after being added, a finding that was also confirmed by our measurements. Since, the experiments didn’t run for a long period of time, the surface tension of the CaCl_2_ solution remained unchanged, thus higher than that of the distilled water even after adding the iron powder.
2. **Calcium Chloride (CaCl**_**2**_**):** Calcium Chloride is highly soluble in water, being a salt, it highly increases the surface tension of water. Another reason behind taking this salt is, its property to increase the temperature of the water after being mixed with water [5], which was used to check whether temperature gradient affects this phenomenon. Throughout this experiment, high-quality anhydrous calcium chloride salt from the company Qualigens, with a minimum assay percentage of 98%, was utilized.
3. **Distilled water:** In this experiment, the distilled water that was used, was produced from a distilled water dispenser, where the electrical conductivity was measured to be 0.04 µ Ss/cm. During our experiment, distilled water of different temperatures was used as well, but for most of the experiments, they remained at room temperature.

The apparatus was designed solely for the purpose of this experiment, it contains a total of, 4 containers, 2 of them were elevated using stool and 2 using retort stands. While one of them was modified to have a channel attached to it, each of them was also modified to have some pipes in them for some necessary purpose.

**Fig 1:**
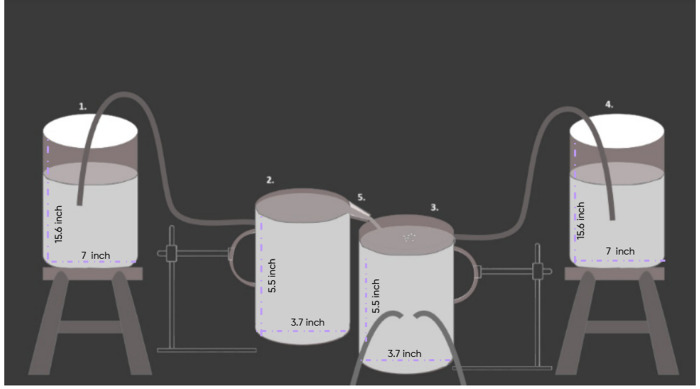
Apparatus.

- **Container 1 & Container 4:** The Containers 1 & 4, respectively labeled as “1” and “4” in the Fig 1, held the capacity to store a total of 10 liters of solution alone. The height of the container and diameter of the base were respectively 15.6 inch and 7 inch.

**Fig 2:**
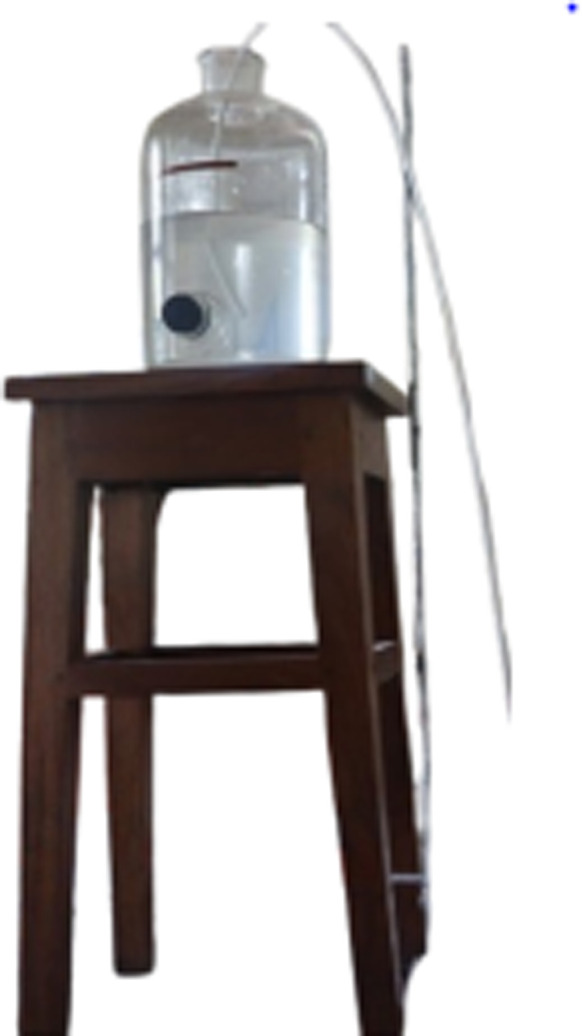
Illustrates the Real life image of Container-4. The experimental setup of container-4 for a better understanding.

Fig 2 shows the real-life image of Container 4, which is elevated on a stool with a pipe emerging from it to transport the stored solution, as shown in the figure. Container 1 follows the same setup.

- **Container 2 & Container 3:** The containers, are labeled as “2” and “3” respectively in the Fig 1. The jars used in the experiment were of the same size, each able to contain one liter of solution. Both of them were kept at the required height using a retort stand. Container 2, was held at a higher height than that of Container 3, so that water could be poured from Container 2, upon Container 3, from the plastic round-line gutter, labeled as “5” in the Fig 1. The containers were made of Polypropylene (PP) plastic, which is considered to be one of the safest plastics [6]. The height of the jar and diameter of the base were respectively 5.5 inch and 3.7 inch.

**Fig 3:**
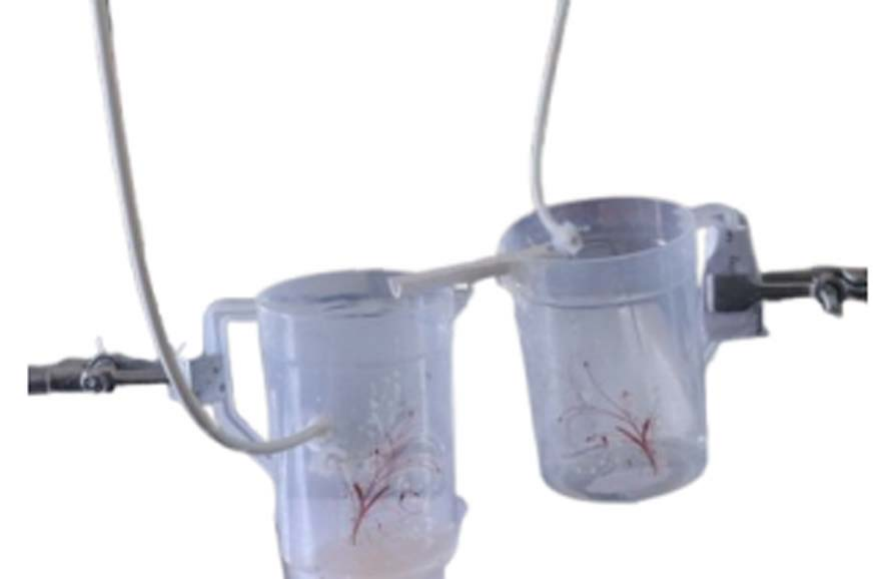
Illustrates the Real life image of Container 2 (in the right) and Container 3 (at the left). The experimental setup of container-2 and container-3, along with the required modifications with pipes and Round-line Gutter.

Below, Fig 3 gives a clear visualization of the main part of the apparatus, showing Container 2 (on the right) and Container 3 (on the left), both positioned at different heights using a retort stand to facilitate the controlled flow of liquid, as shown in the figure.

- **Round-line Gutter:** The gutter used in the experiment was semi-circled, so that water could accumulate in the center before getting poured over the Container 3. It was 2 centimeters in wide and 8 centimeters in length, with a thickness of 1 millimeter. The length and the width of the gutter were respectively 3.5 inch and 0.7 inch.
- **Flexible PVC pipes:** The pipes that were used in the apparatus were made of Polyvinyl Chloride, each of the same 3 mm radius so that the amount of water in Container 2 and Container 3 was kept constant. A total of 4 such pipes were used, where 2 were attached to Container 3, to drain out the solution of it, to avoid the overflow of it. While the third one brings solution from Container 4 to Container 3, the fourth one brings water to Container 2 from Container 1. Each let through 16 ml of the liquids per second.

**Fig 4:**
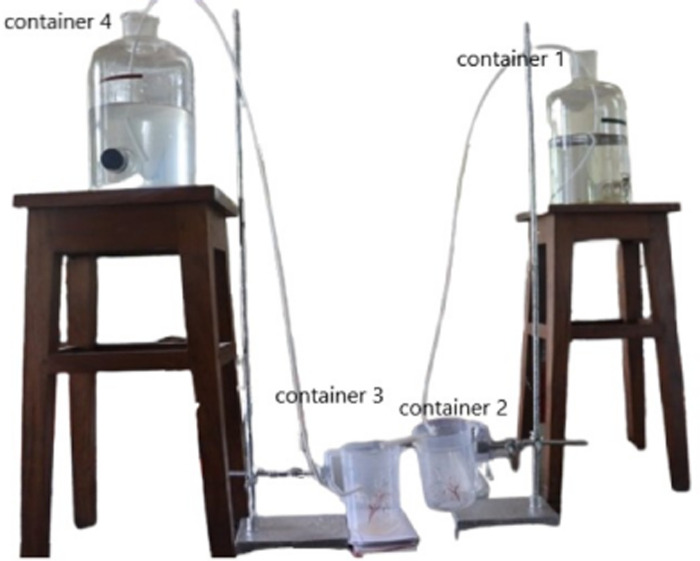
Experimental Setup of the Apparatus. The real life image of the Apparatus is provided for a better understanding.

**Fig 5:**
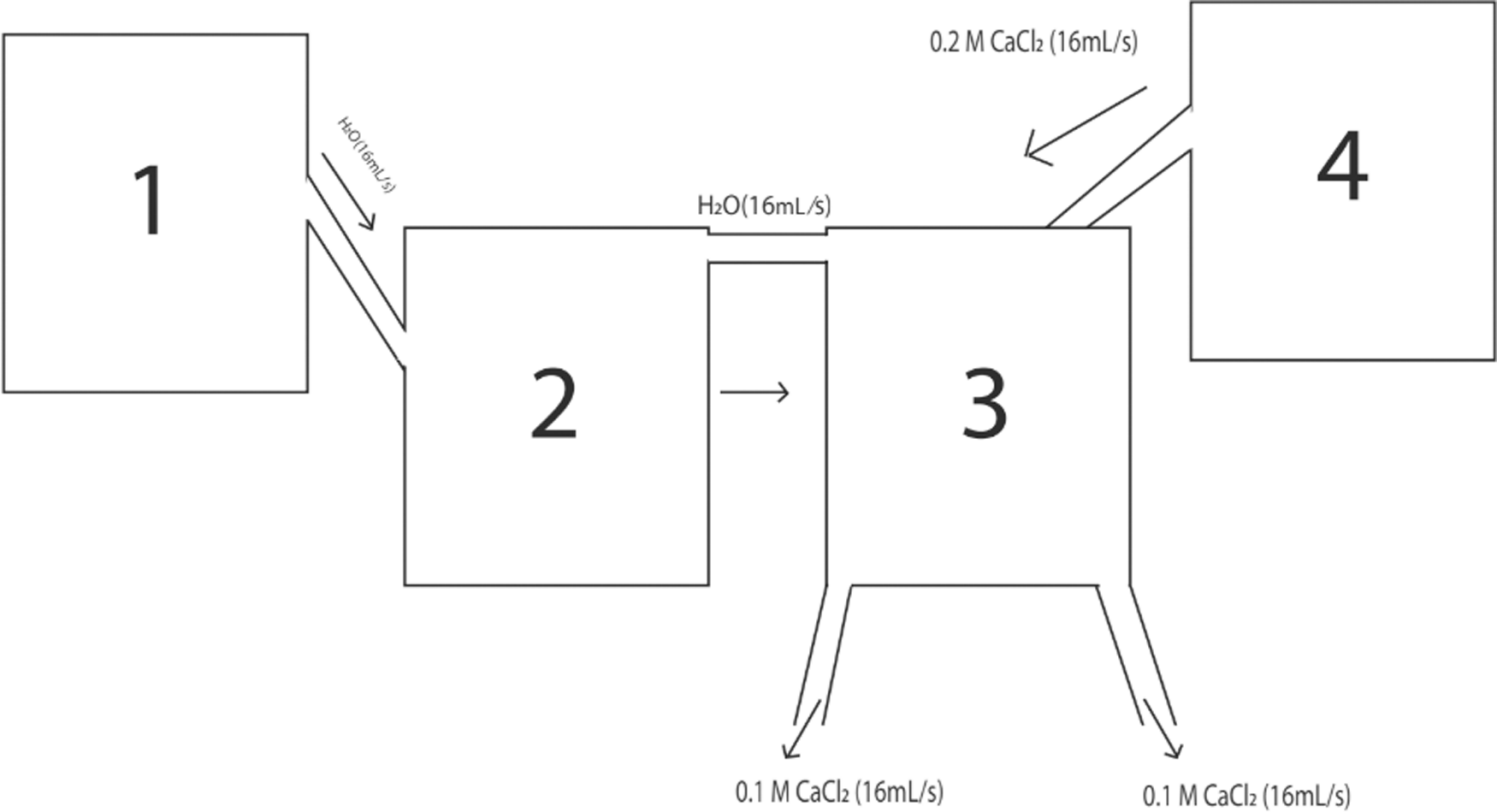
Schematic design of the Apparatus. The schematic design of the Apparatus is provided for a better understanding.

While, Fig 4, helps to visualize the experimental setup of the apparatus, highlighting the connections between the containers, pipes, and other components, the Fig 5 provides a schematic design of the apparatus, providing a detailed layout of the entire experimental system.

## Methodology

### Determination of Surface Tension (5.1)

In the research, for various reasons three different solutions of CaCl_2_ were used, the solutions were 0.1, 0.2 and 0.4 molar aqueous Calcium Chloride. The method which was used to measure the surface tension of the solution was done with the help of capillary action. Where a capillary tube is dipped in the liquid, the height that the liquid rises above the normal surface is taken as the value *“h”* [7].


$$\text{S=}\frac{\text{hρga}}{\text{2cosθ}}$$

This equation was used to measure the surface tension of the solution, where

S, is surface tension of the solution (N/m),

Cosθ, is the contact angle between the capillary tube and the solution,

ρ, is the density of the solution (kg/m^3^)

a, is the radius of the tube (m)

h, is the height of the raised liquid in the capillary tube (m)

g, is the gravitational acceleration (9.8 m/s^2^)

1. The surface tension for normal water was measured to check the accuracy of the instruments. Which presented the exact accurate value, which is 72 mN/m. Later on, the surface tension of 0.1 molar CaCl_2_ solution was measured which presented the equation in the form of-
  $$\text{S}=\frac{0.00712\times 0.0025\times 9.8\times 1008}{2\cos0}$$
2. The density of the CaCl₂ solution after adding 0.1 M CaCl₂ was measured to be 1008 kg/m³, based on a series of careful calculations. The molar mass of CaCl₂ is 110.98 grams, so 0.1 mole amounts to 11.098 grams. When 0.1 mole of CaCl₂ is added to distilled water with a density of 997 kg/m³, the resulting density of the solution becomes 1008 kg/m³. Prior to the addition of CaCl₂, the mass of the solute was accurately measured to the precision of milligrams using a digital weighing scale.
3. Meanwhile, the height of the raised solution was measured to be 0.00712 meters or 7.12 mm and the radius of the tube was measured to be 2.5 mm or 0.0025 meters. While *‘g’*, gives the constant value 9.8 m/s^2^ and Cos0 fetches the value **‘1’**, since the contact angle between any water solution and glass is almost found to be 0°. Thus, the surface tension of the solution takes the value- 0.0878 Newton/meter. Which is 1.2 times the normal distill water.
4. Since 0.2 molar solution was taken in Container 4 for balancing the solution in Container 3 at 0.1 molarity, the surface tension of 0.2 molar solution is not required. But for the experiment in which 0.4 molar CaCl_2_ solution was taken in Container 4, the surface tension did matter. Thus, it was measured in the same way, where the height stood at almost 0.00757 meters and the density took the value of 1041 kg/m^3^. The surface tension was pretty much higher since it was, 0.0956 N/m. Thus, when it was mixed with distilled water in the Container 3 and got reduced to 0.2 molar solution, whatever the surface tension was, it was certainly higher than that of the 0.1 molar solution.
5. The surface tension of 0.1 and 0.4 molar CaCl₂ solutions was measured after sprinkling iron powder on the surface. No changes in surface tension were observed following the addition of the iron powder, which reinforced the reliability of the experimental methodology. This consistency was important, as the lower container consistently showed a higher surface tension than the upper one, which was critical for the success of the investigation. Since the measurements were taken immediately after adding the iron powder, it did not alter the properties of the liquid. The results of the surface tension measurement supported the hypothesis regarding surface tension behavior.

### Experimental investigation on marangoni effect’s solo role (5.2)

To investigate whether the Marangoni effect alone causes the particle movement, the experiment was conducted with a higher surface tension fluid in the lower container (Container 3), and with a lower surface tension liquid in the upper container (Container 2) the conducted experiment followed, the following steps were taken:

1. Container 1, was filled with distilled water, which was later supplied to Container 2 via the PVC pipe.
2. Container 4 was filled with 0.2 molar CaCl_2_ solution, which was sent to container 3 via another PVC pipe.
3. The CaCl₂ solution was introduced into Container 3 when the distilled water began flowing in through the gutter from Container 2. This simultaneous inflow allowed for the adjustment of the solution in Container 3 to achieve a 0.1 molar concentration. The balance was maintained by ensuring that the volume of incoming distilled water matched the amount of CaCl₂ being added through the PVC pipe, facilitating precise control over the concentration of the solution.
4. Once the solution in Container 3 reached the desired height, the two PVC pipes draining the solution were opened to maintain that height. It was carefully ensured that the volume of liquid being drained from these pipes matched the total incoming liquid from both the gutter of Container 2 and the PVC pipe connecting Container 1 to Container 4. This balance allowed for a constant height in Container 3, effectively managing the flow and levels of the connected systems.
5. The prepared iron powder was used to contaminate the surface of the solution of container three through sprinkling over it. Due to the already discussed reasons, a great amount of iron powder floated on the liquid.
6. Then the distill water of the container two was let to get poured over the iron particles which were floating over the CaCl_2_ solution of container 3, in order to observe whether the iron particles floating over the CaCl_2_ solution could ascend the jet of distilled water, coming down through the gutter of container 2.
7. For several times, in between the experiments, the surface was measured to ensure that the surface tension of the lower container remained higher, than the upper one.

### Experimental investigation of the contribution of jet height (5.3)

In this investigation, Container 2 was kept in a such manner so that the gutter remained perpendicular to the vertical axis.

1. At first the gutter was strongly positioned so that no displacement occurred during the investigation.
2. The height of the end-tip of the gutter from different points of container 3 was labeled.
3. The investigation was commenced in the same manner as the experiment described in “5.2”.
4. By continuous closings and openings of the PVC pipes emerging from Container 3, the distance from the end-tip of the gutter and the surface of the solution or, the height of the jet was controlled.

### Experimental investigation of the contribution of the inclined angle (5.4)

In this experiment, the contribution of the inclined angle created between the gutter and the horizontal axis has been studied. The steps that were taken are-

1. Container 2 was inclined by attaching its handle at the required angle along the horizontal axis, with the help of retort stands.
2. For each of the angles, Container 3 was labeled following the set-up of the experiment described in “5.3”.
3. Following it, the experiment was conducted in the same manner as “5.2”.
4. The contribution of each of the angles was measured while keeping the distance between the surface and the end tip of the gutter in notice, to acquire precise results.

### Experimental investigation of the contribution of temperature gradient (5.5)

1. In this investigation, the Container 1 and 4, were filled with liquids of different temperatures, sometimes with liquids of the same temperature and sometimes with liquids having a large gradient between themselves.
2. Later on, the procedure that was followed in 5.3, was implemented in this investigation.

### Experimental investigation of the contribution of the marangoni effect (5.6)

1. The liquids of Container 1 and 4 were reversed, thus the Container 1 was filled with CaCl_2_ solution and the Container 4 with distilled water.
2. Hence, the setup works against the prime setup of our investigation, which later identified the difference in the number of particles going against the stream, between the two setups. Thus, identifying the contribution of the Marangoni effect to the phenomenon of Upstream Contamination.
3. The experiment was later carried on, in the manner of “5.2” but with reversed solutions or surface tensions.

### Experimental investigation of the contribution of the concentration gradient (5.7)

1. The Container 4 was filled with a solution of aqueous Calcium Chloride with the molarity level of 0.4 and a surface tension of 0.0956 N/m.
2. When the solution met distill water coming from the Container 2, in the Container 3, it got balanced and took the concentration of 0.2 molarity. Which still had comparatively higher surface tension than water and the previously used CaCl_2_ solutions.
3. The experiment was later carried out in the procedure following that of 5.2, but with a comparatively higher surface tension than that of the previously used CaCl_2_ solutions.

### Experimental investigation of the contribution of turbulence (5.8)

1. The solution of Calcium Chloride and water in container 4 was mixed with highly purified and non-toxic food-color, meanwhile, the distill water of Container 1, was free of food-colors.
2. Later on, the procedure of “5.2” was followed.
3. In the second go, the distilled water of Container 1 was mixed with food color while normal Aqueous Calcium Chloride Solution was used in Container 4.
4. Afterwards, the same procedure followed in “5.2” was implemented.

### Analysis (5.9)

The entire experiment was recorded using a Canon 250D camera. The recorded videos were later converted into *TIFF* images at a frame rate of 60 frames per second. These images were then analyzed using the software ImageJ and *Fiji*, where adjustments to the brightness and contrast were made. The processed images were subsequently analyzed by comparing the pixel ratios, which identified the moving objects, specifically the iron particles that ascended the jet. The sizes of these particles were measured using a specialized feature that calibrated pixel values to real-world distances, allowing for the approximate determination of the number of particles. But due to the lack of advanced technologies, exact number of particles weren’t achieved, only a approximated value was taken.

## Results and discussion

### Marangoni effect, a contributing factor to upstream contamination

All the experiments that were conducted, revealed that Upstream Contamination is possible without the occurrence of the Marangoni effect, unlike the previous investigations which hypothesized that the Marangoni Effect is solely responsible. In this investigation, container 2 poured distilled water from the gutter attached to it, upon the iron particles floating on the 0.1 molar aqueous Calcium Chloride solution of the Container 3. Where, the surface tension of the CaCl_2_ solution is almost 1.2 times higher than that of normal distill water, thus by no means Marangoni effect can occur here, which clearly states that, whenever there is a surface tension gradient, mass transfer will occur from a lower surface tension region to a higher one [2]. Which restricts the phenomenon of Upstream Contamination *only if the Marangoni effect is the sole factor behind it.*

Meanwhile, our investigations have observed clear signs of Upstream Contamination, where particles from the surface of the 0.1 molar CaCl_2_ solution (Surface Tension = 0.087 N/m) of Container 3 (the lower container), have contaminated the distill water (Surface Tension of 0.072 N/m) of container 2 (the upper container), by climbing up the jet of distill water released by the gutter, which is attached to the Container 2.

### Dependency on the height of Jet (Distance between the End-Tip of Gutter and the Surface of CaCl_2_ solution)

Through the investigation, described in 5.3, a lot of data regarding the dependency of this factor upon Upstream Contamination have been revealed. The particles started climbing up the jet when the height stood at ~ 10 millimeters, and the gutter was inclined perpendicularly to the vertical axis. At the height of 10 mm, no more than 1-2 particles showed up above the gutter, even though lumps of particles were observed accumulating near the jet. When the height was reduced to 2 mm, the number of particles climbing the jet abruptly increased to ~ 8 particles at a time. It hit the threshold of ~ 12 particles when the height was reduced to 1 mm. The observations do perfectly match with the principle of conservation of energy, since the more height it needs to climb, the more potential energy (Mass×Gravitational Acceleration×Height) it needs to achieve, and the more kinetic energy needs to be spent. Thus, the higher the height, the harder to climb and the lesser the number of particles show up.

**Table 1:** Approximated number of particles that ascended the jet at different heights per second.

Height	1 mm	2 mm	4 mm	6 mm	8 mm	10 mm
Number of particles	12	8	5	3	2	1

**Fig 6:**
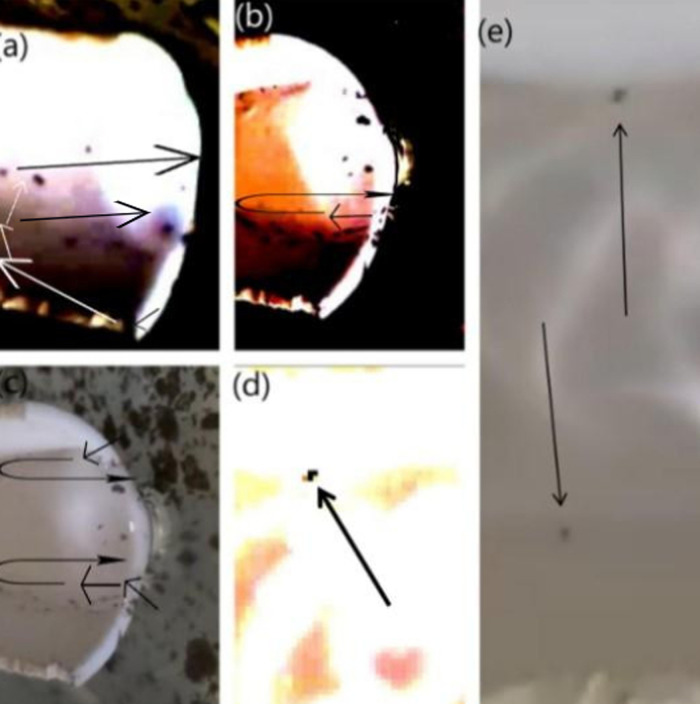
Image of Particles that have ascended different heights. Illustration of upstream contamination occurring without the Marangoni effect, supporting our hypothesis that the Marangoni effect is not the sole factor behind upstream contamination.

**Graph 1:**
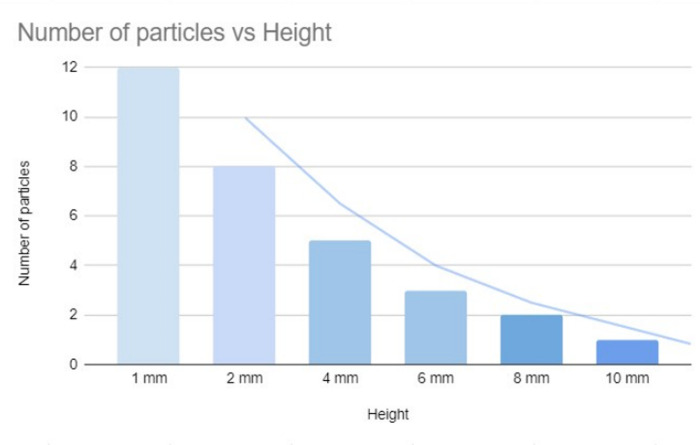
Graphical representation of Table 1. Graphical representation of dependency on the height of Jet, for the ascent of the particles.

At the end of each experimental run, the surface tension of the ferric-contaminated CaCl₂ solution in Container 3 (the lower container) was measured, yielding a value of 0.087 N/m. This value was higher than that of the distilled water in the upper container (container 2), reinforcing the validity of the investigation. The measurement was conducted according to the procedure outlined in section 5.1 of the study. As shown in Fig 6, upstream contamination occurs without the Marangoni effect, supporting our hypothesis that surface tension gradients alone can cause contamination. In Fig 6A, at a height of 2 mm, the number of particles ascending the jet increases. Fig 6B displays a color-contrast adjusted representation of Fig 6C, where at 1 mm height, the number of particles climbing the jet significantly rises. At a height of 10 mm, only a small number of particles are observed, as illustrated in Fig 6D, while Fig 6E shows the results at 8 mm, with fewer particles compared to the 1 mm and 2 mm heights, meanwhile Graph 1, shows how height influences the fluid dynamics and the behavior of particles within the system.

### Dependence on the Inclination and establishment of a New Hypothesis

The experiments described in 5.4, have brought such facts, which hint toward a new hypothesis, *dependence on the Boundary Layer Condition and dependence on the exerted force upon the contaminants through the jet, due to buoyancy force and the drag force.* The angles that were checked in the investigation of 5.4, perfectly blended with the hypothesis. The angles that were examined are-

1. **Gutter aligned to the vertical axis Perpendicularly or horizontally 0°:**

This angle showed the least number of particles going against the stream, which perfectly agrees with the boundary layer effect. Because at this angle, there is no vertical component of the water’s motion, thus the thickness of the boundary layer is the maximum, thus the velocity of the water jet observes a gradual slope, which causes the differences in the velocity of the water stream near the walls and the middle to be very low. Thus, the particles get a very low amount of advantage near the walls because the force exerted as drag force upon the particles is low as well, due to a small gradient. The results of this investigation and the results of investigation 5.3 are the same because it was also done by setting the gutter perpendicularly to the vertical axis [3].

2. **Gutter aligned to the horizontal axis at 25°:**

This investigation showed an increased number of particles going against the stream, which approves the hypothesis once again. At 25°, it acquires a vertical component, which works with the gravitational force, thus the liquid’s velocity, which is coming down from the middle increases by a great portion, but the liquid near the walls doesn’t increase that much due to skin friction. Thus, the particles experience a stronger drag force than that of 0°, due to the thinner boundary layer [8].

**Fig 7:**
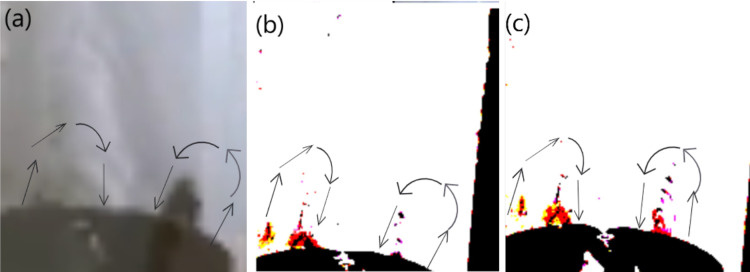
Image of Particles which have boarded the 25° inclined-gutter after climbing different heights. (A) Particles ascending a 25° inclined gutter at 7 mm height. (B) Particles at 4 mm height. Fig 7C: Particles at 2 mm height.

The observation that is captured in the panel (a) is peculiar indeed, but it was observed after the creation of a disturbance in the solution, to break down the lumps of iron powders that formed due to the existing cohesive force between those particles. Thus, through the observations made in Section 5.4, the increased particle ascent at a 25° inclination supports the hypothesis of boundary layer effects and drag force variation (see Fig 7A-C).

3. **Gutter Inclined at 45° Angle:**

**Fig 8:**
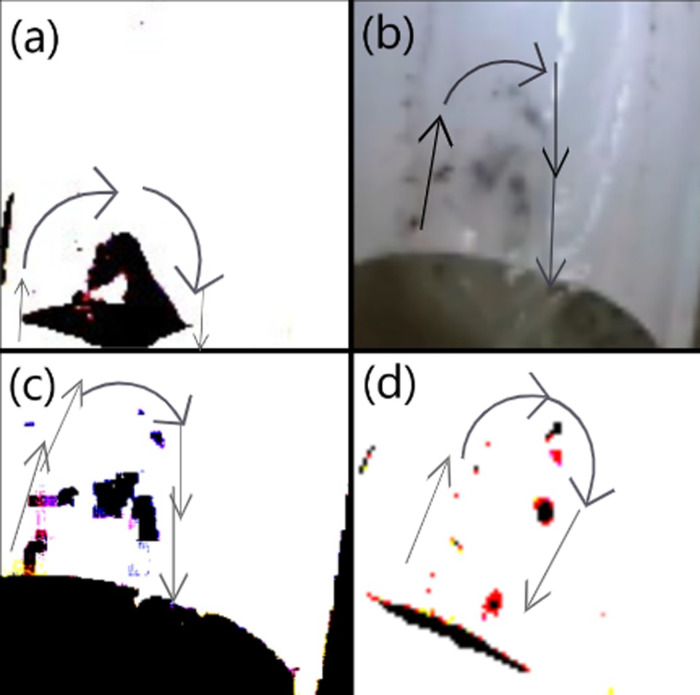
Image of Particles which have boarded the 45° inclined-gutter after climbing different heights. Particles ascending a 45° inclined gutter at various heights: (A) at 1 mm, (B) at 3 mm, (C) is a color-contrast adjusted image of panel B, and (D) at 2 mm.

As a follow-up to the investigation with the gutter set at a 25° inclination, this study observed that the number of particles rising against the stream was significantly greater at a 45° inclination compared to both 25° and 0° inclinations. The reason behind this is similar to that of the 25° inclination, at 45°, the water stream experiences equal vertical and horizontal components of force. This balance increases the velocity gradient between the middle and the border of the liquid, creating a greater drag force, thus resulting in a greater number of particles ascending through the jet as shown in Fig 8A-D below:

4. **Gutter Inclined at 60° Angle:**

The results of this investigation, at first seemed incompatible with the previous results, but later on, it perfectly coincided with the hypothesis regarding the Boundary Layer Effect.

In accordance with the investigations that were conducted, taking the inclined angle as 25° and 45°, this experiment should have given a greater number of particles, because the vertical component is much greater. But the results were something else, the number of particles that climbed up the jet was no greater than that of 45°, but lagged behind by a small margin.

**Fig 9:**
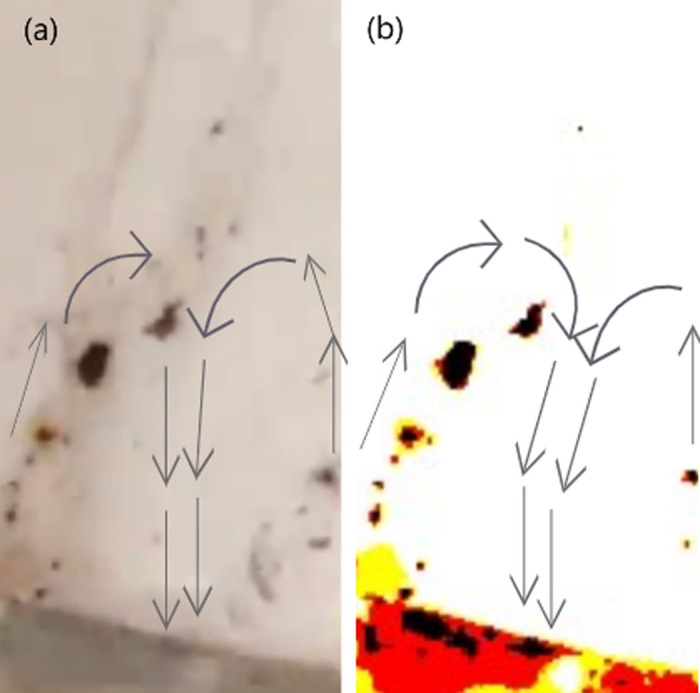
Particles ascending a 60° inclined gutter at different heights: (A) at 2 mm and (B) is a color-contrast adjusted image of panel A to enhance particle visibility.

But after considering all the factors of the Boundary Layer Effect, it is deemed to be all right, because at 60° inclination, the horizontal component decreases by a huge factor, resulting in a decreased shear force. Which causes lesser skin friction near the walls, thus a decreased velocity gradient [3,9] as shown in Fig 9A-B below:

5. **Gutter Inclined at 90° Angle:**

As hypothesized, the number of particles that climbed the gutter inclined at 90° was significantly small but still greater than that of 0°. Because when it is inclined vertically, it loses all of its horizontal component. Leading to a small shear force and skin friction, thus a thicker boundary layer and a small velocity gradient. Again, due to the sudden separations between the wall and the fluid, it sometimes becomes turbulent which makes the number of particles rising to be significantly higher than that of normal 0° inclination [3].

**Fig 10:**
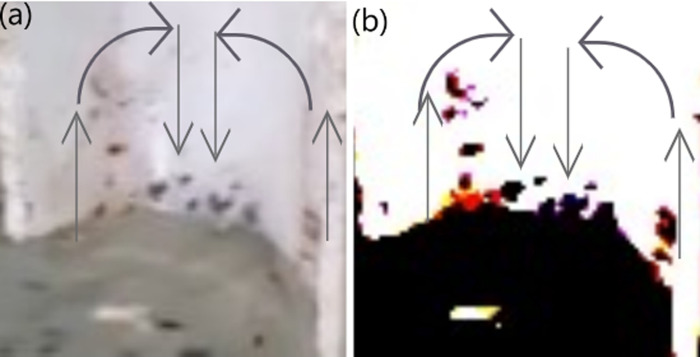
Image of Particles which have boarded the 90° inclined-gutter after climbing different heights. Particles ascending a gutter inclined at 90° at different heights: Panel A shows particles at 2 mm height, and Panel B provides a color-contrast adjusted version of panel A to better visualize the particles.

Through the analysis of the results, of the angle-focused experiments, we have once again proved our main hypothesis that the Marangoni effect is only a supporting factor because, in none of these experiments, it had been in an effect. Again, these experiments have established a new hypothesis, that drag force, buoyancy force, and the boundary layer effect are the key role-players of the phenomenon. Because the number of particles that went against the stream at 45° was at a peak, meanwhile the number started decreasing while the angle was set far from 45° whereas, the number was higher at 90° than that of 0°. As a matter of excitement, all these correspond with the Boundary Layer Effect.

**Table 2:** Approximated number of particles that could climb up the gutter (per second), after going through jet of 2 mm while the gutter was inclined at different angles.

Angle	0°	25°	45°	60°	90°
Number of particles	8	14	21	15	12

**Table 3:** The final analysis of particle behavior based on gutter angle inclination.

Angle	Vertical Component of Velocity	Horizontal Component of Velocity	No-Slip Boundary Layer	Drag Force
0	None	Fully Horizontal	Thickest boundary layer	Minimal Drag Force
25	Greater Vertical component than 0 but lesser than 45	Lesser Horizontal Component than 0 but greater than 45	Thinner than 0	Greater Drag force than 0 but lesser than 45
45	Equal Vertical and Horizontal Component	Equal Vertical and Horizontal Component	Thinner than 25	Strongest Drag Force
60	Greater Vertical Component than 45 but lesser than 90	Lesser Horizontal Component than 45 but greater than 90	Thicker than 45	Lesser Drag Force than 45 but greater than 90
90	Fully Vertical Component	None	Thick but due to sudden separations between the fluid and the walls, a sudden velocity gradient is created.	Very low Drag Force, but greater than 0

**Fig 11:**
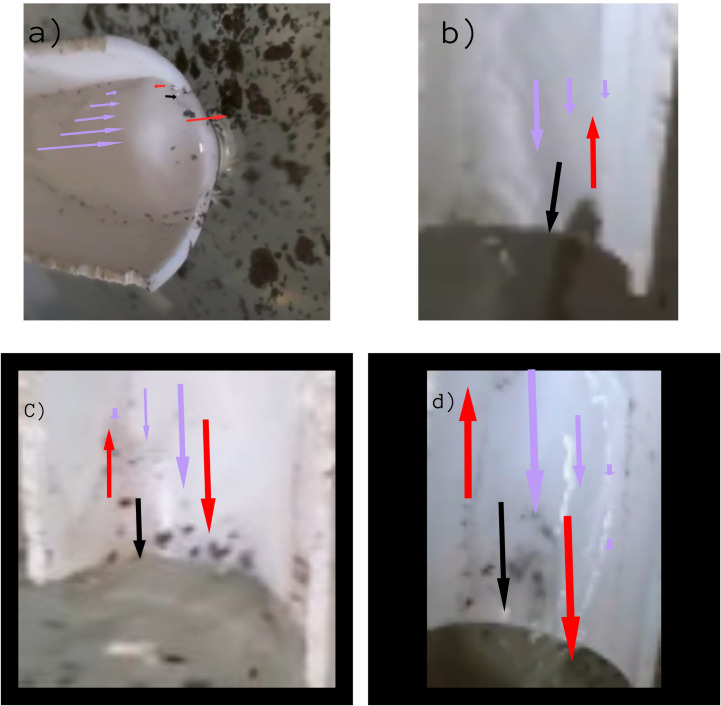
Illustration of particles interacting within a gutter inclined at various angles. The figure depicts: (1) varying downstream water velocities caused by skin friction (indicated in purple), (2) drag forces exerted on upstream particles by nearby downstream water (indicated in black), and (3) particle motion (indicated in red). The arrow sizes provide a comparative visualization of the respective velocities and forces; however, the ratios between the arrows do not represent actual magnitudes. Note that the black arrows indicate the direction of the nearby water stream and not the direction of the drag force; the drag force acts opposite to the black arrows. Figure illustrates particle interactions in gutters inclined at different angles: Panel A (0°) shows a gradual velocity increase from the wall, panel **B** (25°) demonstrates a faster velocity rise due to a weaker no-slip effect, panel **C** (90°) shows a similar behavior to 0°, and panel **D** (45°) displays the largest velocity increase and abrupt separations due to the weakest no-slip condition.

From the figures, it is evident that the size of the purple arrows near the wall is smallest, indicating that due to skin friction, the velocity at the wall is effectively zero. As the water stream moves away from the wall, its velocity increases. This increase is gradual in:

- Fig 11A (0°): Gradual velocity increase with the strongest no-slip condition.
- Fig 11B (25°): More pronounced velocity increase due to a weaker no-slip effect.
- Fig 11C (90°): Similar gradual increase as in 0°, with a slight variation in flow direction.
- Fig 11D (45°): The most pronounced velocity increase, with the weakest no-slip condition and abrupt separations from the wall.

These variations are due to the interplay of vertical and horizontal velocity components and the gravitational effects on the water stream.

**Graph 2:**
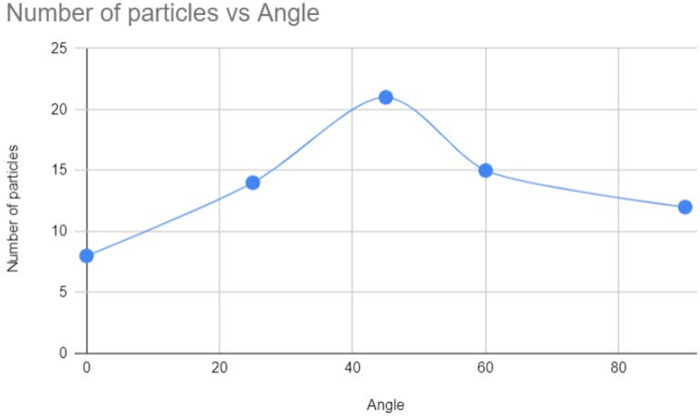
Graphical Representation of Table 2. Graphical representation on the dependency of the gutter-inclination for the upstream ascent of the particles.

Since particles tend to ascend within portions of the water stream where velocity is lowest, the drag force will be greatest in scenarios where the boundary layer is thinnest and the velocity gradient is steepest. A steeper velocity gradient near ascending particles causes faster-moving water streams to exert a stronger drag force, resulting in a greater number of ascending particles with higher velocities. Consequently, particle ascent is maximum for 45°, lower for 25°, 90°, and 60°, and lowest for 0°. The Graph 2, completely illustrates how a 45-degree angle maximizes particle ascent, demonstrating the optimal angle for fluid movement.

### Independence of Temperature, and Temperature Gradient (Difference of Temperature between the liquids of Upper and Lower Container)

This conclusion was reached after several different experiments. Liquids of different temperatures were taken in containers 1 and 4, ranging from 20°-85° Celsius. The exothermic property of Calcium Chloride was also considered to ensure the balance of temperature between the liquids. The experiments conducted in this range saw no difference from any other experiment conducted at other temperatures in this same range.

Some experiments were also done while maintaining large temperature differences between the liquids of containers 1 and 4, the experiments were conducted in such a manner, that in some experiments, one of the containers witnessed itself as hotter while the other one was cooler, and during other experiments itself as cooler and the other one as hotter. In these experiments, no abrupt differences were observed in accordance with the experiments that held other heat-based conditions. This once again supports the hypothesis, that in this phenomenon Boundary Layer Effect is involved. Since, whenever temperature changes, the viscosity of the whole fluid changes, causing the thickness of the boundary layer to remain the same since the velocity of the fluid changes as a whole with an equal factor. Even though a boundary layer named thermal boundary layer is formed, which is related to heat flow, causing no effect on the particle movement [3]. Thus, no difference in the particle movement is seen in the phenomenon of Upstream Contamination.

### Independence of Concentration Gradient

Through the experiment described in 5.7, a big concentration gradient between the two containers was set. This concentration gradient also resulted in a larger surface tension gradient between the two containers. Unlike, other experiments where the lower concentration had a greater surface tension than the upper one by a factor of “1.2”, this experiment observed the lower container to have greater surface tension by a factor of “1.3”. Thus, the Marangoni effect has no chance to play a role in this experiment just like all the other experiments conducted [10], except the one described in “5.6”. Even though, this experiment also observed the phenomenon of Upstream Contamination, asserting the claim of this experiment once again, that the Marangoni Effect is only a supporting factor and not the sole cause.

The number of particles that ascended through the channel was no greater or no lesser than that of other experiments, which supports our claim that concentration gradient doesn’t matter for the phenomenon of upstream contamination when the particles are going from a higher surface tension region to a lower surface tension region.

### No Visible Evidence of Turbulence’s Role

The experiments conducted with food-colors observed no uncommon patterns, only the normal phenomenon of Upstream Contamination was observed. But due to lack of sufficient data, no conclusion regarding this question of Turbulence, can be reached.

### Contribution of Marangoni Effect in a Normal Scenario

Through the experiment described in 5.6, it was clearly observed that the Marangoni Effect indeed is a supporting factor to the particles climbing up to the gutter against the stream. This particular experiment set the gutter at the 45° inclination, which showed notable differences from the experiments that didn’t let the effect occur. With the support of the Marangoni Effect, particles were able to climb up almost 13 mm jet, while in normal experiments it started climbing up from 10 mm. Again, the investigation with the Marangoni effect enhanced the number of particles going against the stream to a great extent. The above data has been presented below through the Table 4.

**Table 4:** Approximate number of particles ascending to the gutter of Container-2 per second at varying heights, with the container inclined at 45°, both with and without the influence of the Marangoni Effect. For the 13 mm jet, the number of particles was 43 with the Marangoni Effect and 0 without it. For the 2 mm jet, 21 particles ascended with the Marangoni Effect, while no particles ascended without the Marangoni Effect. The Marangoni Effect was induced by adding CaCl^₂^ to the higher container through Container-1.

Height	13 mm	2 mm
With Marangoni Effect	5	43
Without Marangoni Effect	0	21

**Fig 12:**
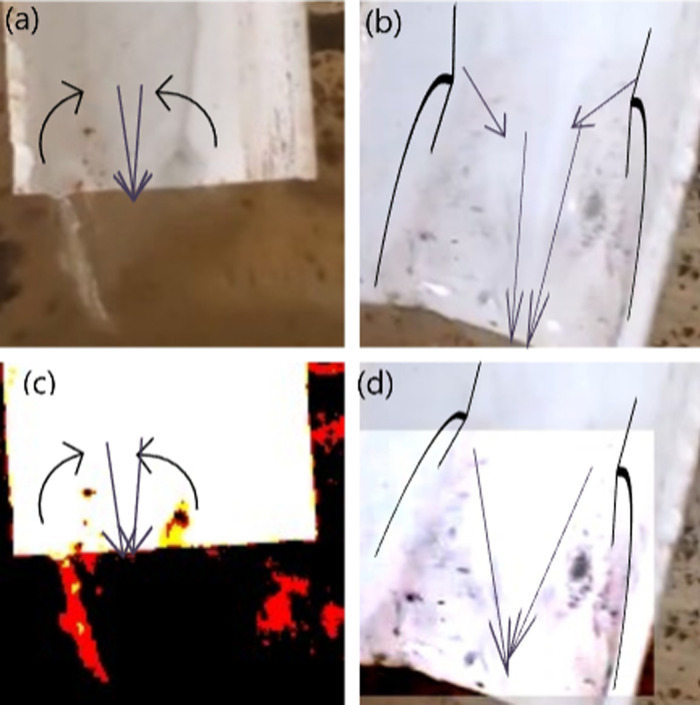
Image of Particles which have boarded the 45° inclined-gutter after climbing different heights, in the presence of the Marangoni Effect. Fig. 12 shows particles ascending a 45° inclined gutter with the presence of the Marangoni Effect at different heights: Fig. 12A shows particles at 13 mm, Fig. 12B at 2 mm, Fig. 12C is a color-contrast adjusted version of Fig. 12A, and Fig. 12D is a color-contrast adjusted version of Fig. 12B.

**Graph 3:**
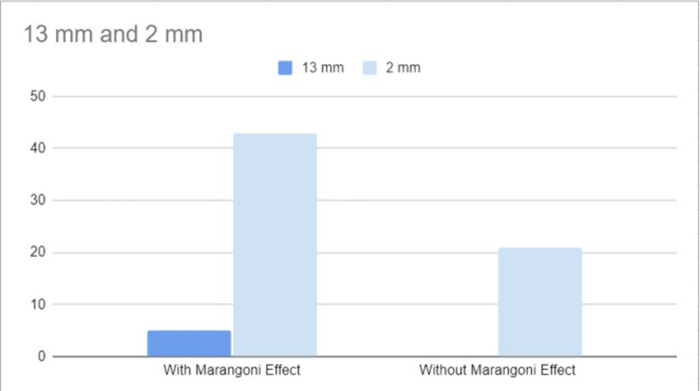
Graphical Representation of Table 4 The Contribution of Marangoni Effect, in the particle ascent has been visualised in the Graph 3.

Thus, the observation that is presented with Graph 3, is another support to our claim because it proves that while the effect takes place, the particles do get an advantage, but they can also accomplish the same without the effect but with a harder approach, which causes a decreased particle number.

### Contaminant’s physical properties

Throughout the experiments, a very peculiar thing was observed, the particles tend to go against the downstream water-fall more, whenever they are more finely grounded. This highlights another possible cause for the phenomenon since very small iron particles can float above the surface of the solution due to solution’s surface tension and due to microscopic bubble formations between the pores, these bubbles may cause a buoyant force to act over the particles which makes it climb up the jet [4].

This hypothesis clearly explains that why the experiments that used less grounded particles, showed lesser contamination. Because bigger particles’ weight exceeds the surface tension of the solution which causes it to sink. At the same time, bigger particles tend to have lesser air pockets, thus fewer bubbles. And since these bubbles might have been helping the particles to climb up the jet with the buoyancy force, a lesser number of them may cause lesser climbing.

An argument might be raised that, bigger particles shall have lesser air pockets but will be bigger in size, so why wouldn’t they ascend in the equal amount? Since the particles will have a rougher surface and have more weight than the supporting surface tension of the solution, big bubbles wouldn’t be enough to help.

#### Vortexes in the Jet and the Gutter.

Just like the experiments that were carried out by the previous researchers, our experiments have also observed the same phenomenon. The particles were found to get stuck in somewhat a vortex while climbing up the Jet and also faced a vortex while being on the Gutter. In each and every investigation they were found to rise from the border and return through the middle.

**Fig 13:**
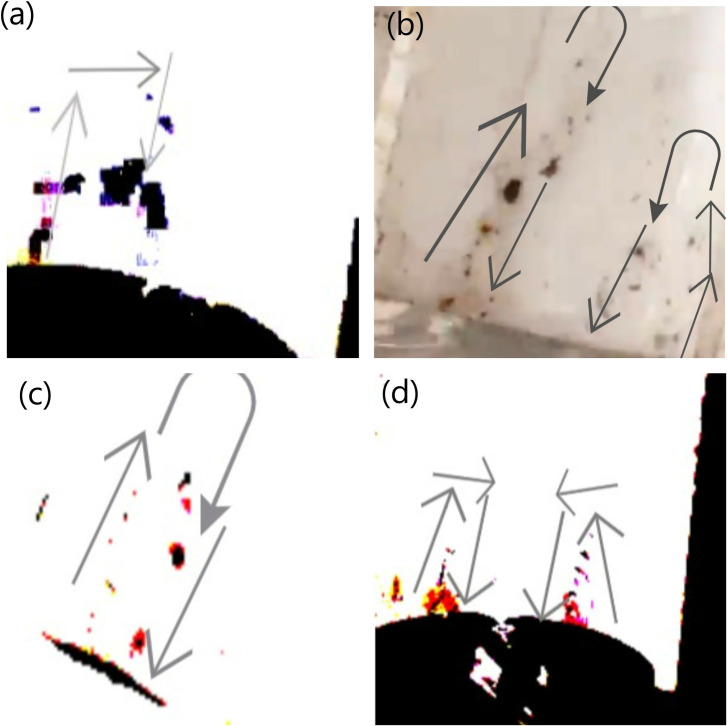
Image of Particles in Vortexes, which have boarded the inclined-gutter after climbing different heights. Particles in vortexes ascending gutters inclined at different angles and heights: panel A shows a 3 mm jet in a 45° inclined gutter, panel B shows a 4 mm jet in a 45° inclined gutter with the Marangoni Effect, panel C shows a 2 mm jet in a 45° inclined gutter, and panel D shows a 2 mm jet in a 25° inclined gutter.

This finding supports our claim of the effective role of Boundary Layer Condition because in that effect fluid going through the middle remains faster and thus stronger than that of the fluid near the horizon because the fluid near the horizon stays at a “no-slip condition” due to the skin-friction along with the walls. Thus, when the water jet coming from the gutter hits the iron powder floating on the solution of container 3, the particles get shifted towards the horizon, since they experience lesser force over there. And then due to the constant force exerted by the surrounding faster water stream and due to the buoyancy force acting over them due to in-between air pockets, they rise through the stream. Again, due to the Boundary Layer Condition, the water stream beside the particles, which is faster than the water stream through which they are rising, a drag force is exerted over them, which supports them in their ascend through the jet of water [11,12], this can be clearly visualized through Fig 13.

Our research has identified potential indications of vortex formation, possibly generated by the drag force acting on the particles, which could contribute to the development of a Kamran vortex street. This phenomenon provides an explanation for the continuous ascent of particles in a sequential manner within the vortices. As a particle ascends against the liquid, it creates disturbances in the fluid flow, leading to the formation of a Kamran vortex street that reduces the resistance encountered by subsequent particles, facilitating their ascent. Supporting this hypothesis, we observed that when a particle ascended the jet, other particles followed almost instantaneously along the same path, behaving as if connected in a chain-like manner as shown from Fig 13A-D. This behavior suggests that the ascending particle creates a localized void along its path by penetrating through the jet near the channel walls, which reduces the viscous forces acting on the particles and enhances their collective ascent. These observations reinforce the idea that vortex-induced disturbances play a crucial role in facilitating and accelerating particle movement within the flow [13].

## Conclusion

Through a set of meticulously planned investigations into the phenomenon of Upstream Contamination, a lot of new pieces of information have been achieved. All indicate some new physics.

The particle movement from the higher surface tension solution of the lower container to the lower surface tension liquid of the higher container has proved the hypothesis that the Marangoni Effect, is a mere supporting factor behind the particles’ ascend through the jet. Further experiments have also shown that a large number of particles can ascend a jet of a greater height if the effect is in play, but without the effect, it ascends comparatively a lower height, in a smaller number. However, the height at which the particles can ascend without the support of the Marangoni Effect is not small either.

Through a series of experiments regarding particle nature, vortexes, temperature, concentration gradient, and others, we have been able to establish a new hypothesis that, particles ascend the jet primarily with the help of the buoyancy and the drag force. The drag force is exerted on the particles due to some specific *no-slip Boundary Layer Conditions*, which is supported by the investigations that we have conducted until now. But to prove this hypothesis regarding the buoyancy and the drag force, more research has to be done considering the effective role of the Boundary Layer Effect. Although this still remains a hypothesis due to lack of accurate measurement of the number of particles, it has been well established by our approximated results.

In conclusion, we have not only proven that this phenomenon is possible even without the effective role of the Marangoni Effect but also established a new hypothesis that is supported by our results. Future investigations should be carried out to delve into this, for the crucial understanding of the driving forces behind this captivating phenomenon.
